# Preparation of Cu/Fe-NaA Zeolite Catalysts from Coal Gangue for High-Efficient Fenton-like Catalytic Degradation of Basic Magenta

**DOI:** 10.1021/acsomega.4c07931

**Published:** 2025-02-05

**Authors:** Naren Tuya, Liming Qi, Chengyue Dong, Sitong Lan, Sijia Zhang, Lixiang Wang, Xiaoli Wang

**Affiliations:** Inner Mongolia Key Laboratory of Environmental Chemistry, College of Chemistry and Environmental Science, Inner Mongolia Normal University, Hohhot 010022, P.R. China

## Abstract

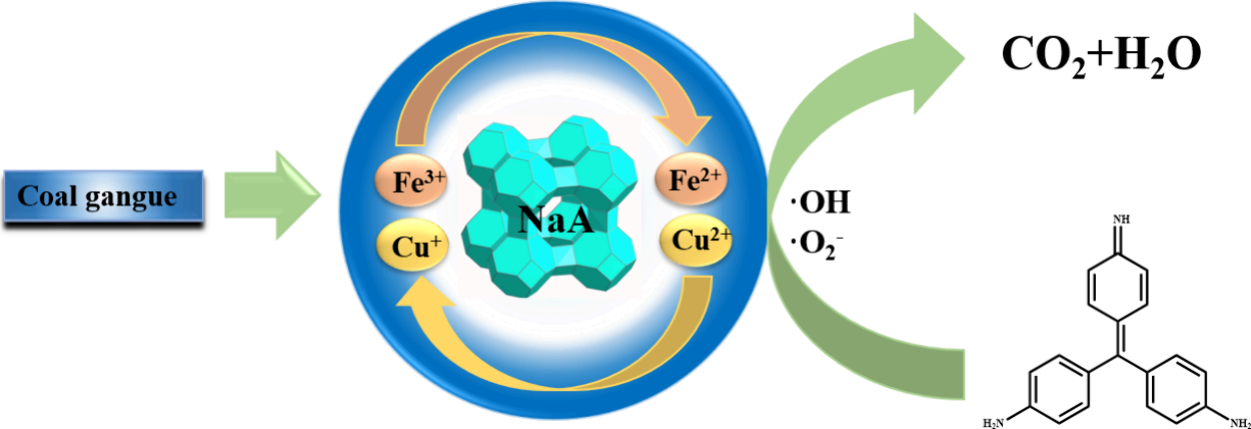

A highly efficient and stable copper and iron loaded NaA zeolite catalyst (Cu/Fe-NaA) was successfully prepared from Inner Mongolia gangue through an impregnation method, and it exhibited a great removal rate for basic magenta dye from drain water solution. Basic magenta dye is a widely used dye in the textile industry. However, the organic compounds and pigments produced during the production and use of the dye cause pollution and harm to the environment and human beings. The results showed that Cu/Fe-NaA had good degradation and decolorization activities and stability for alkaline magenta dye, and the catalytic degradation rate of the alkaline magenta solution could reach 99.84% in 30 min. Therefore, Cu/Fe-NaA exhibits great potential for the degradation of organic dyes to realize environmental governance.

## 1 Introduction

Coal gangue is a solid waste discharged in the process of coal mining and physical separation that is mixed with coal seams and constitutes about 10∼15% of coal production, and it is harder than coal with a lower carbon content.^1^ A large amount of coal gangue piled up for a long time will cause environment pollution. So, it is an urgent task to find some solutions to consume the coal gangue. In the gangue, the total amount of SiO_2_ and Al_2_O_3_ reaches up to 65∼95%, which can act as the silicon and aluminum sources of zeolite, and that make it possible to utilize coal gangue as the raw material for synthesizing zeolite. Zeolite, a type of molecular sieve, is a porous aluminum silicate crystal with excellent adsorption and ion-exchange capabilities and is commonly used for chemical separation, environmental manipulation, and catalytic response and in materials industry. Among them, sewage treatment is a crucial field, and zeolite molecular sieves play an important role in treating pollutants in water.^2^

Basic magenta is an organic dye frequently used in industrial fields like textile and dyeing.^3^ The presence of dyes in the environment, even in low concentrations, is harmful to marine organisms and inhibits their growth. For this reason, various water treatment technologies such as filtration, disinfection, and coagulation have been developed to remove dyes from wastewater. Among them, chemical oxidation is one of the most effective and least expensive techniques to remove organic compounds.^4^ Currently, heterogeneous Fenton catalysis is widely used as one of the advanced oxidation processes for treating organic dyes in wastewater.^5^ Compared with homogeneous Fenton catalysis, heterogeneous Fenton catalysis has the advantages of higher utilization efficiency of H_2_O_2_, wider pH range,^6,7^ and without the production of a large number of iron sludge and iron salts.^8^ Zeolite molecular sieves have great potential as catalyst carriers because of their low cost, environmental friendliness, and large specific surface area.^9^ After loading transition metal ions, the modified zeolite can catalyze H_2_O_2_ to generate reactive radicals, which can degrade organic pollutants.^10,11^ Besides, zeolite, a heterogeneous Fenton catalyst, also shows great advantages in recycling and separation.^12,13^

In this study, highly efficient and stable copper and iron loaded NaA zeolite catalysts (Cu/Fe-NaA) were successfully synthesized from the Inner Mongolia coal gangue through the impregnation method. Copper and iron with the advantages of low cost, low toxicity, and higher activity^14^ can catalyze the degradation of hydrogen peroxide to generate hydroxyl radicals^15^ which can promote catalytic activity. The catalytic degradation rate of Cu/Fe-NaA to alkaline magenta is up to 99.84% at 30 min. After recycling, the degradation rate of Cu/Fe-NaA can also reach 94.72%. Therefore, it provides a choice and great potential to degrade organic dyes to realize environmental governance.

## 2 Experimental Section

### 2.1 Materials

The gangue used in this experiment was from the Xuejiawan area, Ordos City, Inner Mongolia. Cu(NO_3_)_2_·3H_2_O (AR), Fe (NO_3_)_3_·9H_2_O (AR), basic magenta (C_20_H_20_CIN_3_), hydrochloric acid (AR), isopropyl alcohol (IPA, C_3_H_8_O) (AR), and KI (AR) were purchased from Sinopharm Chemical Reagent Co, China. Hydrogen peroxide H_2_O_2_ (30 wt %) (AR), p-benzoquinone (BQ, C_6_H_4_O_2_) (AR), and NaN_3_ (AR) were purchased from Tianjin Damao Chemical Reagent Factory, China. Anhydrous ethanol (AR) was purchased from Tianjin Zhiyuan Chemical Reagent Co.

### 2.2 Preparation of Cu/Fe-NaA Catalyst

#### 2.2.1 Preparation of NaA Zeolite

The composition of the gangue from the Xuejiawan area is shown in Table 1. The content of SiO_2_ and Al_2_O_3_ accounts for 80.98% of the total content, and the molar ratio of SiO_2_ and Al_2_O_3_ can be calculated as 1.95, which provides a good condition of the silica–aluminum ratio for the synthesis of the molecular sieve of NaA zeolite. Since the contents of Fe_2_O_3_, CaO, MgO, K_2_O, and Na_2_O are rare, the influence of them on the experiment is negligible.

**Table 1:** Major Contents of the Raw Coal Gangue from Xuejiawan Area (By % in Weight Oxides)

sample	SiO_2_	Al_2_O_3_	Fe_2_O_3_	CaO	MgO	K_2_O	Na_2_O
content (%)	43.21	37.77	0.22	0.066	0.046	0.11	0.243

In this experiment, the NaA zeolite was synthesized in sodium hydroxide system through the hydrothermal synthesis method, with coal gangue in the Xuejiawan area as the raw material. The coal gangue was roasted at 550 °C for 2 h to decarburizate and then cooled to room temperature for use. 2.000 g of pretreated coal gangue was weighed, the alkaline-ash ratio was adjusted to 1.2, the silica–alumina ratio was adjusted to *n*(SiO_2_):*n*(Al_2_O_3_) = 2:1, and then the sample was roasted in a muffle furnace at 750 °C for 2h. After that, sodium hydroxide was mixed with the above product with ratios of *n*(NaOH):*n*(SiO_2_) = 2:1 and *n*(H_2_O):*n*(NaOH) = 30:1, stirred at 25 °C for 12 h, and then crystallized at 80 °C for 24 h. At last, the product was washed with distilled water to pH < 11 and dried to obtain NaA zeolite.

#### 2.2.2 Preparation of Cu/Fe-NaA Zeolite Catalyst

**Scheme 1:**
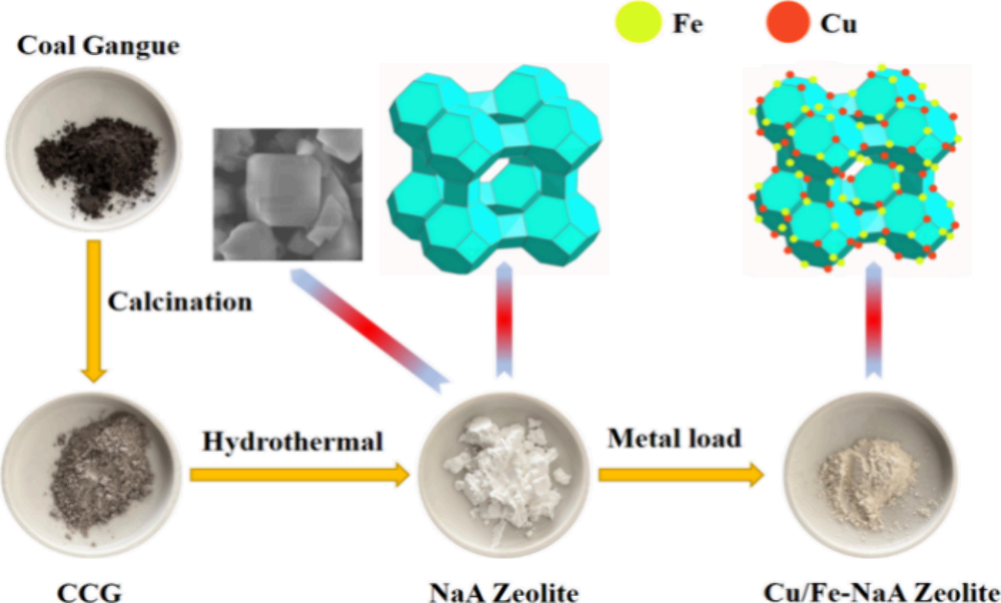
Preparation of the Cu/Fe-NaA Zeolite Catalyst

Cu/Fe-NaA zeolite catalysts were prepared by the impregnation method. NaA zeolite was added to different concentrations of Fe(NO_3_)_3_·9H_2_O and Cu(NO_3_)_2_·3H_2_O solutions, stirred at room temperature for 2 h, and then sonicated at 25 °C for 30 min to promote the loading of iron to the NaA zeolite. After that, the suspension was crystallized at 80 °C for 6 h. At last, the Cu/Fe-modified NaA zeolite catalysts were obtained after washing and flitering. The procedure is shown in Scheme 1. Four different ratios of Cu/Fe-modified NaA zeolite catalysts (Cu/Fe-NaA) were prepared and named as 0.5 g/L Cu/Fe-NaA, 0.8 g/L Cu/Fe-NaA, 1.0 g/L Cu/Fe-NaA, and1.5 g/L Cu/Fe-NaA, respectively.

**Scheme 2:**
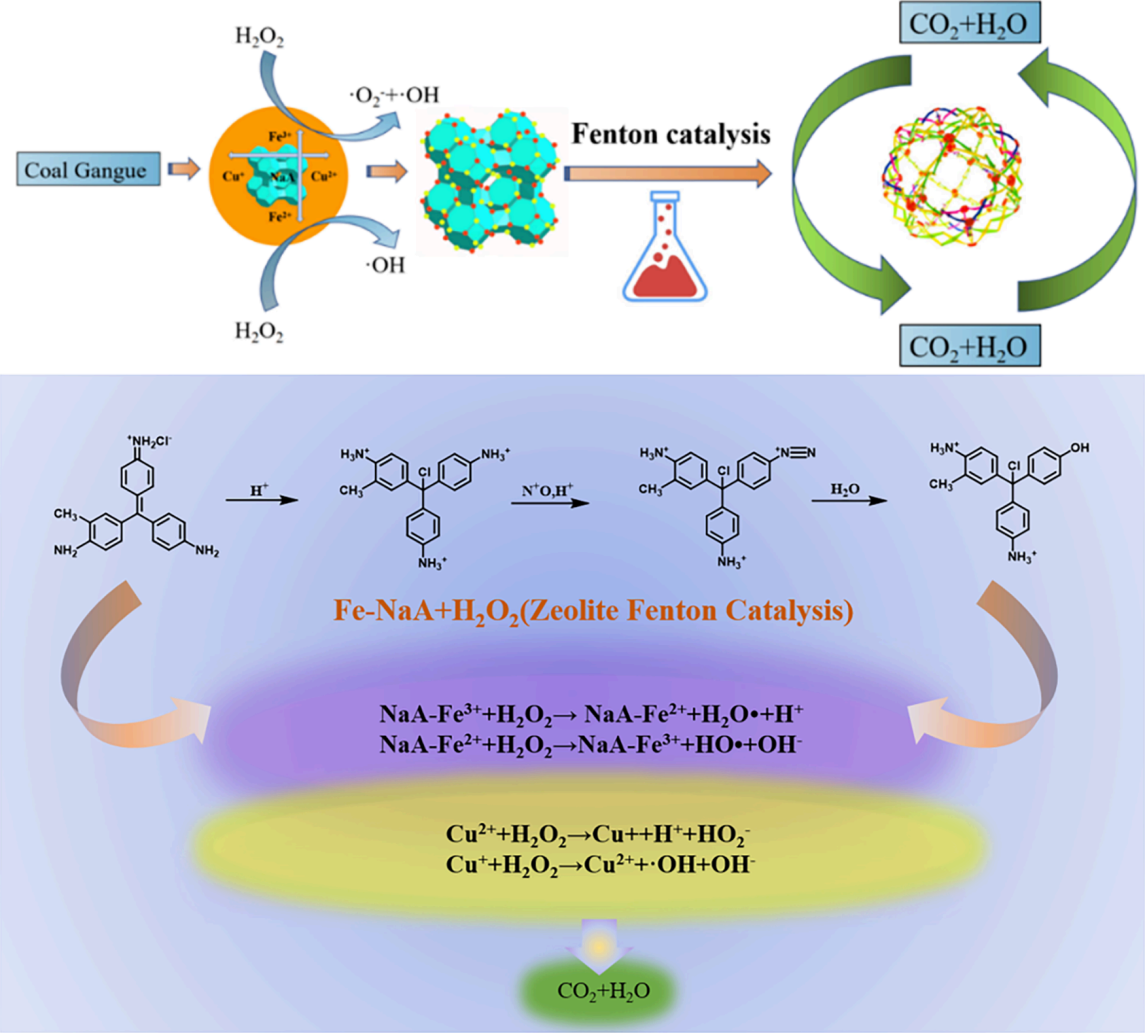
Possible Mechanism of Catalytic Degradation of Basic Fuchsin by Fenton-like Reaction on Cu/Fe-NaA

### 2.3 Heterogeneous Fenton-Catalyzed Degradation of Basic Magenta

The catalyzing process was proceeded by adding 0.6000 g of Cu/Fe-NaA zeolite catalyst and 1.5 mL of 30% H_2_O_2_ into the basic magenta solution and stirring at room temperature. The influence of the concentrations of Fe^3+^ and Cu^2+^, the quantity of the Cu/Fe-NaA zeolite catalyst, the temperature, the initial pH of the solution, the initial dye concentration, and the concentration of H_2_O_2_ on the degradation efficiency of basic magenta were explored through monitoring photoabsorption at different times by a UV–vis spectrophotometer. The obtained values of photoabsorption were substituted into the standard curve to calculate the concentration of the degraded basic magenta solution, and the degradation rate (η, %) was calculated by the following formula η = (*C*_0_ – *C*_t)_/*C*_0_ × 100%, where *C*_0_ and *C*_t_ are the concentrations of basic magenta in the solution at the initial and t moments, respectively. The possible catalytic mechanism was deduced.

### 2.4 Characterizations

An UItima IV powder X-ray diffractometer (Rigaku Corp.) was used to analyze the forms of components in samples. The specific surface area and pore size distribution of the samples were determined and analyzed using an ASAP 2020 pore structure specific surface area analyzer (American Micromeritic Company). A 6700 Fourier infrared spectrometer (Nicolet Company, USA) was used to analyze the species of the component and function group on the surface of the catalyst. The surface morphology of materials was analyzed by using a SU 4800 scanning electron microscope (Hitachi). X-ray photoelectron spectroscopy (XPS) was performed with monochromatized A1X-ray and Kα-radiation (*h*υ = 1486.6 eV) using a ThermoFisher K-a spectrometer (ESCalab250xi) manufactured by Thermo Fisher Scientific, USA. Multi

An N/C 3100 TOC analyzer (Jena Analytical Instruments, Germany) was used to measure the total organic carbon (TOC) content before and after catalytic degradation. A Bruker EMXplus-6/1 electron paramagnetic resonance spectrometer (ESR, Germany Electronics Company) was used to analyze the ·OH and superoxide radical (·O_2_^–^) captured by DMPO. The photoabsorption of the degraded dyes was measured using a UV-5100 UV–visible spectrophotometer manufactured by Shanghai Yuan Analytical Instruments Co.

## 3 Results and Discussion

### 3.1 XRD Analysis

**Figure 1:**
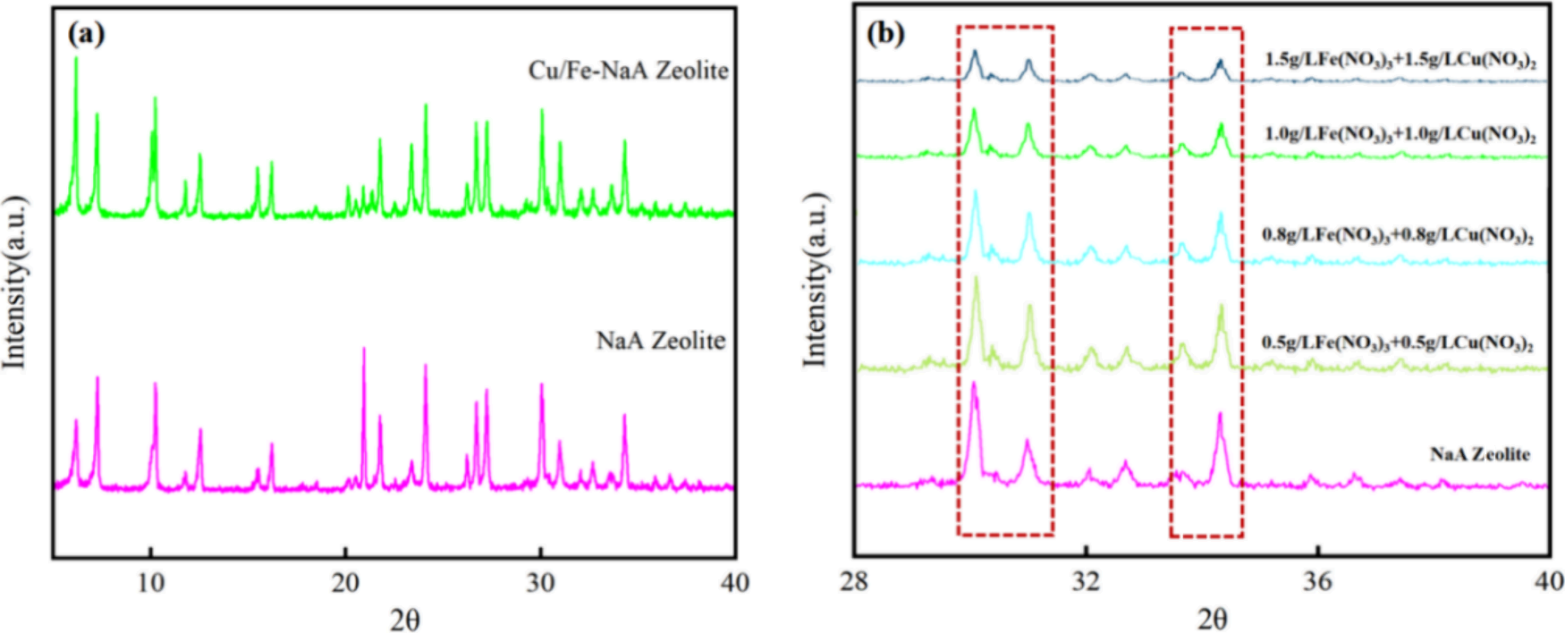
XRD patterns of NaA zeolite and Cu/Fe-NaA zeolite (a) and Cu/Fe-NaA zeolite with different Cu/Fe contents (b).

X-ray diffraction (XRD) analysis of Cu/Fe-modified NaA zeolite and pristine NaA zeolite is shown in Figure 1. There was no significant change in the peak location of Cu/Fe-modified NaA zeolite, and the characteristic diffraction peaks appeared at 2θ of 7.28, 10.26, 20.92, 24.06, 33.52, and 34.48°, which is in high agreement with the pristine NaA zeolite. It means that Cu/Fe-modified NaA zeolite had the same structure as pristine NaA zeolite. The characteristic peaks of Cu/Fe-NaA zeolite catalysts decreased when the concentrations of Cu^2+^ and Fe^3+^ were gradually increased^16^; according to the Bragg equation 2dsin θ = *n*λ, the shift of the diffraction peaks to a lower angle indicates that the cell of Cu/Fe-NaA is enlarged,^17^ but it did not affect its structure. According to the literature, Cu and Fe are distributed on the surface of the catalysts in a highly uniform dispersion and mainly in the form of Fe^3+^, Cu^2+^, and metal-oxide particles.^4^

### 3.2 SEM-EDS Analysis

**Figure 2:**
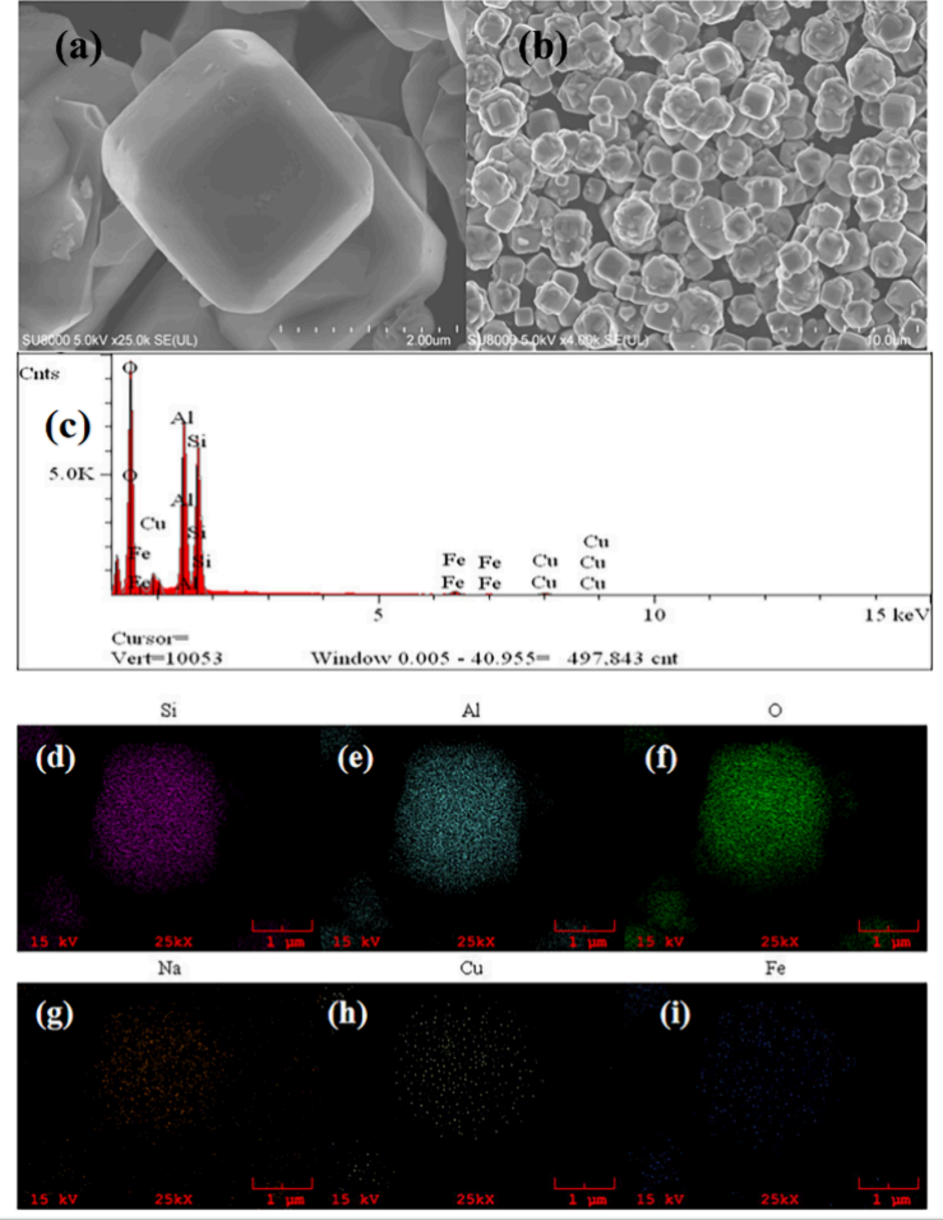
SEM spectra of (a) NaA and (b) Cu/Fe-NaA. (c) EDS spectrum of Cu/Fe-NaA. (d–i) Mapping image of Cu/Fe-NaA.

Scanning electron microscopy (SEM) images of pristine NaA zeolite and Cu/Fe-NaA zeolite catalyst are shown in Figure 2a,b, respectively. It can be found that the structure of pristine NaA zeolite is mainly ortho-hexagonal crystals. However, the appearance of small particles on the surface of the Cu/Fe-NaA zeolite^18^ may be attributed to the loading of Cu/Fe on the surface of the zeolite, whose morphology is the same as that of the pristine NaA zeolite, suggesting that the loading of the transition metal has not changed the structure of the zeolite molecular sieve. Figure 2c shows the EDS spectra of Cu/Fe-NaA, confirming the successful loading of Cu/Fe onto CG. Figure 2d–i shows the element distribution image of Cu/Fe-NaA, indicating that Si, Al, O, Na, Cu, and Fe are uniformly distributed in the material.

### 3.3 BET Analysis

The calculating results of Brunauer–Emmett–Teller (BET) surface structural parameters of NaA zeolite and Cu/Fe-NaA catalyst are shown in Table 2. NaA zeolite molecular sieve is an excellent carrier material for catalyst synthesis, and its enriched specific surface area provides an active site for the dispersion of Cu and Fe. The effect of the initial concentration of Cu^2+^ and Fe^3+^ on the surface area of the catalyst was studied. When the concentration of copper and iron was lower than 1.0 g/L, the specific surface area of NaA zeolite is nearly not influenced, and the specific surface areas of 0.5 g/L Cu/Fe-NaA, 0.8 g/L Cu/Fe-NaA, and 1.0 g/L Cu/Fe-NaA were 16.3251, 15.7022, and 16.3006 m^2^/g, respectively. When the concentration of iron was more than 1.0 g/L, the specific surface area and pore volume of the catalyst decreased significantly,^19^ the specific surface area of the sample 1.5 g/L Cu/Fe-NaA was just 9.0994 m^2^/g, and its corresponding pore volume showed a significant decrease. This may be caused by the lower crystallization of the 1.5 g/L Cu/Fe-NaA catalyst or the lower distribution uniformity of the metal, and the excess metal ions caused accumulation to block some of the original pores of the NaA zeolite.

**Table 2:** Surface Structure Parameters of Fe-NaA Zeolite

sample	specific surface area /(m^2^/g)	pore volume /(cm^3^/g)
	*S*_BET_	*V*_total_
NaA	15.8024	0.083
0.5 g/L Cu/Fe-NaA	16.3251	0.089
0.8 g/L Cu/Fe-NaA	15.7022	0.080
1.0 g/L Cu/Fe-NaA	16.3006	0.091
1.5 g/L Cu/Fe-NaA	9.0994	0.079

### 3.4 FT-IR Analysis

**Figure 3:**
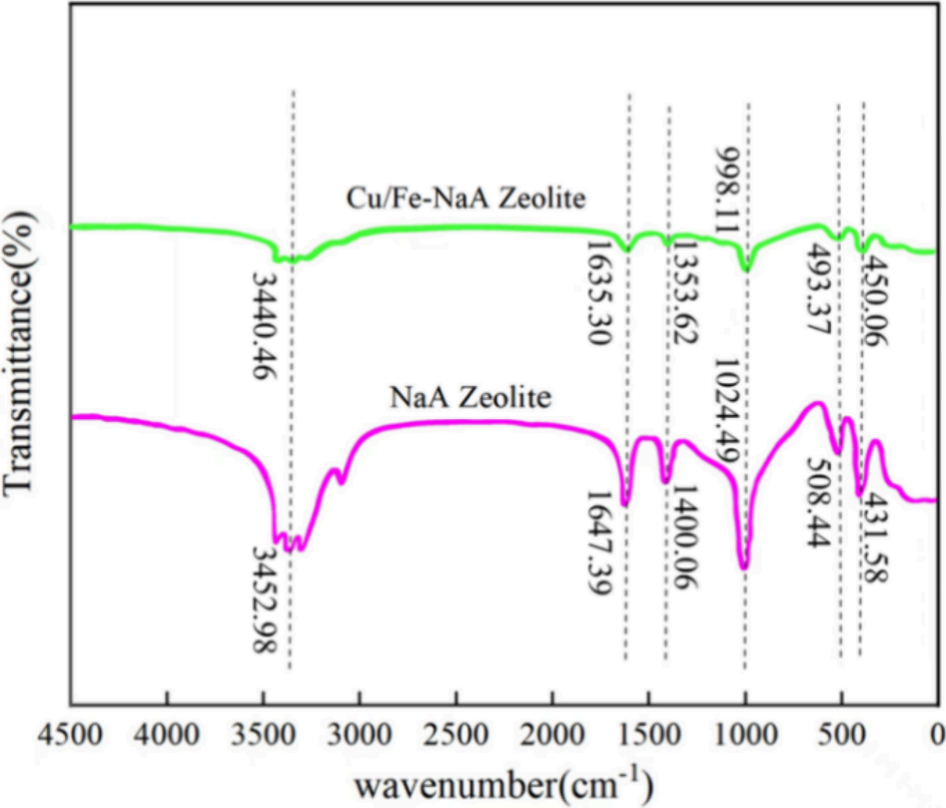
FT-IR patterns of NaA and Cu/Fe- NaA zeolite.

Fourier transform infrared (FT-IR) spectra of the Cu/Fe-NaA zeolite catalyst and NaA zeolite are shown in Figure 3. The peaks at 3440.46 and 1635.30 cm^–1^ are caused by the stretching and bending vibrations of the H–O bond of the zeolite, respectively. The peak at 998.11 cm^–1^ is stronger, and it is due to the antisymmetric stretching vibrations of the Si–O bond. The band appearing around 400–800 cm^–1^ is due to the vibrations of Si–Al along the external of the crystal. It is noteworthy that the peaks at 800 and 400 cm^–1^ have mainly been caused by both the vibrations of the (Si/Al)–O bond. Compared with NaA zeolite, the characteristic peaks of the Cu–Fe-loaded zeolite are shifted toward lower wave numbers. This is due to the fact that the introduction of metal ions can change the infrared spectrum of the zeolite depending on its mass, charge, ionic size, and cationic environment.

### 3.5 XPS Analysis

**Figure 4:**
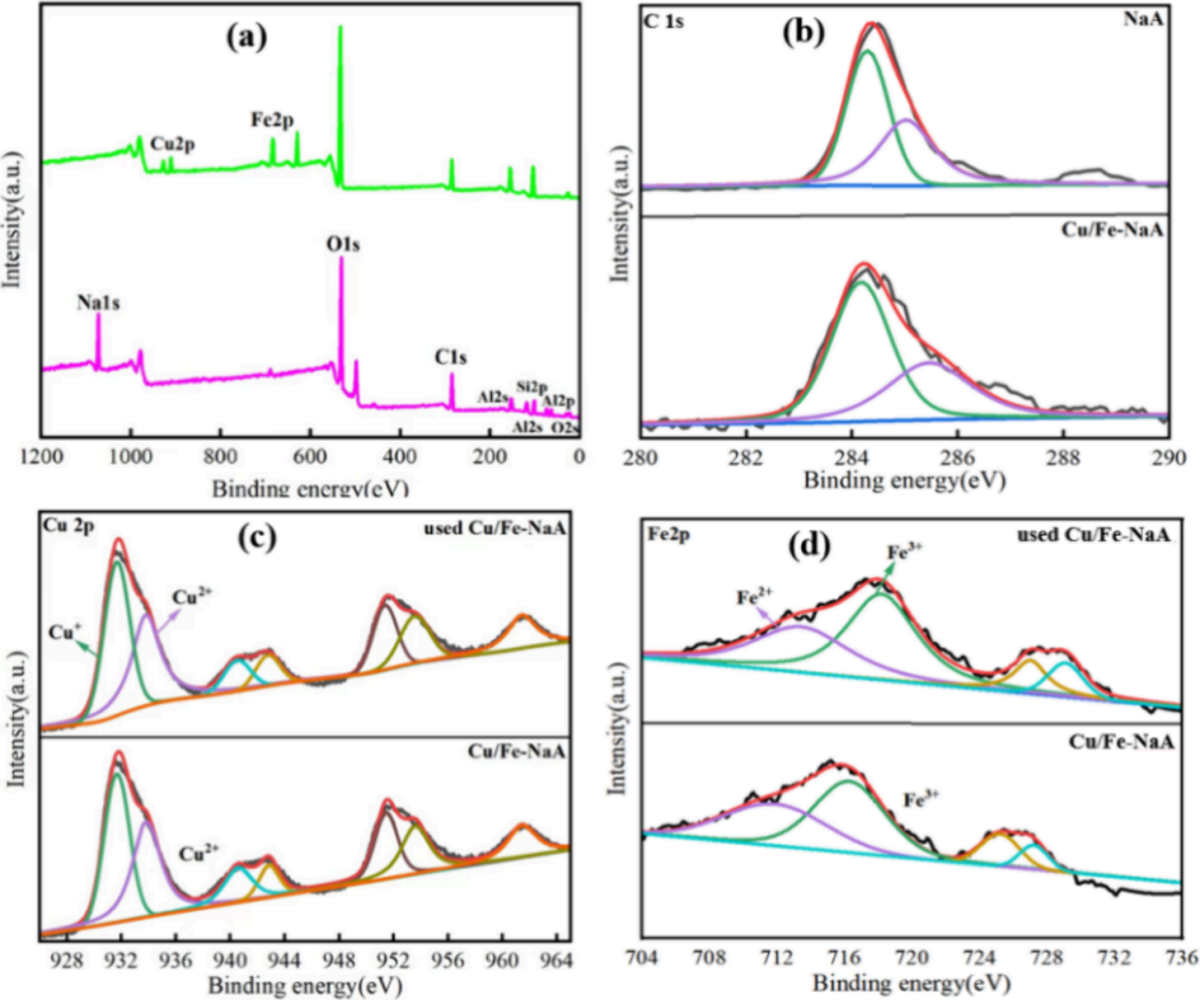
XPS spectra of different samples: (a) Zeolite XPS total spectrum. High-resolution XPS spectrum: (b) C 1s, (c) Cu 2p, and (d) Fe 2p.

Figure 4 shows the XPS spectra of the pristine NaA and Cu/Fe-NaA catalysts; the investigated spectra show significant binding energy peaks for the elements Cu 2p, Fe 2p, O 1s, and C 1s (Figure 4a). There are significant binding energy peaks for the elements C 1s, Cu 2p, and Fe 2p (Figure 4b–d). It is highlighted that the newly added Cu loading was proved in Figure 4c. The Cu 2p3/2 XPS spectrum of the sample has three pairs of peaks at 928–960 eV, and the peak at 932.2 eV is due to the presence of reduced Cu species. The peak at 942.5 eV represents the formation of Cu–O–SiO,^5^ and 952 eV represents satellite peaks.^20^ The deconvolution analysis showed that the Cu species in the catalyst is mainly in the form of copper oxide and Cu^2+^. The peaks^21^ observed at 710.5 eV (Fe 2p3/2) and 724.7 eV (Fe 2p1/2) corresponded to Fe 2p (Figure 4d), and a satellite peak oscillating at 718.3 eV was also observed, confirming the presence of iron oxide in the Cu/Fe-NaA catalysts.^22^ High-resolution XPS analysis showed that the multivalent cycling of Fe and Cu in the Cu/Fe- NaA catalyst was very favorable for the activation of H_2_O_2_, the redox potentials of Fe^2+^ and Fe^3+^ were higher than those of Cu^0^ or Cu^1+^ and Cu^2+^, and the Cu ions could promote the cycling of Fe^3+^ to Fe^2+^ to further facilitate the decomposition of Fe^2+^ acting on H_2_O_2_ to generate ·OH.^23^

### 3.6 Catalytic Performance and Mechanism of Cu/Fe-NaA Zeolite

#### 3.6.1 Effect of Cu/Fe Ion Concentration

**Figure 5:**
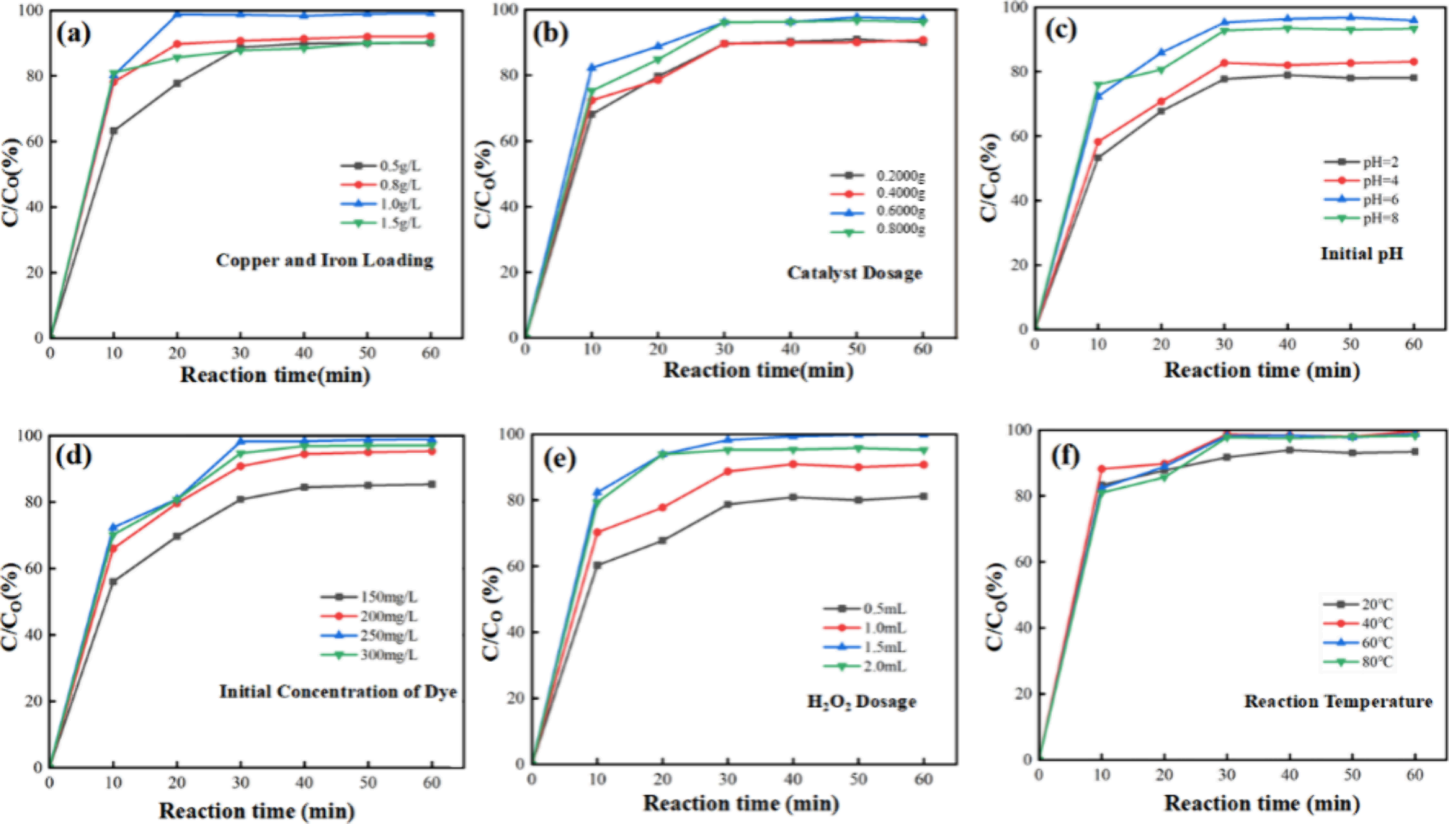
Influence of various parameters on the degradation rate of basic magenta: concentration of Cu^2+^ and Fe^3+^ solutions during ion exchange. (a) copper and iron loading; (b) catalyst dosage; (c) initial pH; (d) initial concentration of dye; (e) H_2_O_2_ dosage; (f) reaction temperature.

The participation of iron can greatly improve the degradation efficiency of basic magenta. The effect of Cu/Fe ion concentration in the range of 0.5–1.5 g/L on the degradation efficiency of basic magenta was explored in Figure 5a. The highest degradation efficiency appeared at the concentration of 1.0 g/L, and the degradation rate was 99.11%, which was attributed to the powerful adsorption capacity of the zeolite molecular sieve, which could increase the active site to capture the dye molecules on the surface of the catalysts and thus promote the degradation efficiency. However, when the concentration of copper and iron ions reached 1.5 g/L, the removal efficiency was reduced. This may be because the limited pore fusion volume of the zeolite molecular sieves was blocked by excessive copper and iron ions.^24^ So, 1.0 g/L was selected as the optimal copper and iron ion concentration.

#### 3.6.2 Effect of Cu/Fe-NaA Catalyst Dosage

The effect of Cu/Fe-NaA zeolite catalyst dosage on the degradation efficiency is shown in Figure 5b; with the increase of the amount of catalyst, more adsorption sites and adsorption surface area were provided to capture the basic magenta, so that its removal rate gradually increased. With the increase of catalyst dosage, the degradation efficiency reached the maximum in 30 min and then remained unchanged. When the amount of dye cations is constant, the sustaining increased dosage of the catalyst will cause a waste. So, 0.6000 g was chosen as the optimum Cu/Fe-NaA zeolite dosage.

#### 3.6.3 Effect of Initial pH of Solution

The initial pH of the solution also affects the rate of decomposition of surface-charged H_2_O_2_ and of the catalyst. Therefore, we investigated the effect of pH of the initial solution on the degradation efficiency of basic magenta, and the results are shown in Figure 5c, which reveals that the degradation efficiency of basic magenta is very sensitive to pH, and it reached about 80% at pH = 2 and 4, and with the increase of pH, it can be maximized within 30 min at pH = 6. The degradation efficiency of basic magenta slightly decreased at pH = 8. The possible reason is that under alkaline conditions, H_2_O_2_ decomposes rapidly to O_2_^25^ instead of generating ·OH, so that the catalytic activity decreased, and therefore, the optimum pH was chosen as 6.

#### 3.6.4 Effect of Initial Concentration of Alkaline Magenta

The effect of basic magenta solution concentration on the degradation efficiency was investigated, and catalytic degradation was carried out at different initial basic magenta solution concentrations (150–300 mg/L). The results are shown in Figure 5d. With the increase of the initial concentration of basic magenta, more basic magenta molecular was captured by the catalyst, so the reaction rate gradually increased, and the maximum degradation efficiency reached at 30 min when the initial concentration was 250 mg/L. However, with sustaining increase of the initial concentration to 300 mg/L, the degradation efficiency shows a decreasing tendency, which is due to the fact that with the initial solution concentration increase, the catalyst may be affected by basic magenta and reaction products,^26^ leading to a decrease in its stability and thus affecting the degradation efficiency. Hence, 250 mg/L was selected as the optimal initial concentration of basic magenta.

#### 3.6.5 Effect of H_2_O_2_ Dosage

In addition, the H_2_O_2_ dosage also plays an important role in the Fenton-catalyzed process. In other words, the Fenton-catalyzed degradation of basic magenta was controlled by the synergistic catalytic effect of Cu/Fe species and H_2_O_2_. Figure 5e shows the effect of H_2_O_2_ dosing (0.5–2.0 mL) on the degradation efficiency of basic magenta. The results showed that the degradation efficiency increased from 81.18 to 99.89% with the increase of H_2_O_2_ dosing from 0.5 to 1.5 mL, which was attributed to the increase of ·OH produced by H_2_O_2_ in the solution.^27^ However, when the H_2_O_2_ dosage was increased to 2.0 mL, the degradation efficiency decreased to 95.20%. The decrease in the degradation performance may be related to the extra amount of H_2_O_2_ reacting with reactive hydroxyl radicals to generate hydroperoxyl radicals (·HO_2_), and therefore, 1.5 mL was chosen as the optimal amount of H_2_O_2_ dosing for the reaction system.

#### 3.6.6 Effect of Reaction Temperature

The effect of temperature on the catalytic performance was investigated, and the results are shown in Figure 5f. When the reaction temperature reaches 40 °C, the degradation efficiency is significantly increased, which is because of the rate of ·OH generation at low temperatures; when the reaction temperature increases, the ·OH generation rate is accelerated, improving the catalytic activity. It basically remained unchanged after the temperature was further increased, and the degradation rate reached close to 100% in 30 min. Therefore, 40 °C was selected as the optimum temperature for the degradation of basic magenta by Cu/Fe-modified NaA zeolite catalyst.

### 3.7 Catalytic Mechanism

**Figure 6:**
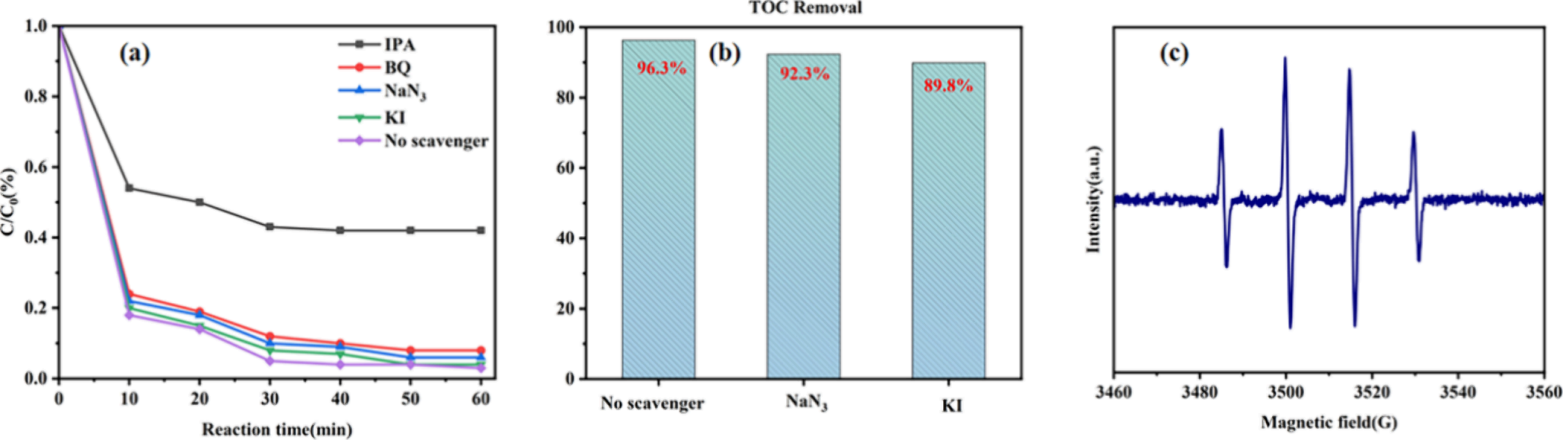
(a) Effect of IPA, BQ, NaN_3_, and KI on the basic magenta degradation efficiency. (b) Effect of NaN_3_ and KI on the removal efficiency of TOC. (c) ESR spectrum of the DMPO-·OH Cu/Fe-NaA + H_2_O_2_ system.

In order to determine the reactive species produced during the nonhomogeneous Fenton catalysis of the Cu/Fe-NaA catalyst, the common free radical inhibitors IPA, BQ, NaN_3_, and KI were used to trap the hydroxyl radicals (·OH), peroxyl radicals (·O_2_^–^) produced during the oxidation process,^28^ single linear oxygen (^1^O_2_), and holes (h^+^).^29^Figure 6a shows that only the addition of IPA significantly inhibited the degradation of basic magenta. In the presence of IPA, the removal of basic magenta decreased from 98.6 to 58%. Unusually, the basic magenta removal efficiency did not change significantly with the addition of the other free radical inhibitors, and these results suggest that the active species produced in this nonhomogeneous Fenton-like system is mainly ·OH.^30^ To further verify the radical species, ESR analysis of the Cu/Fe-NaA + H_2_O_2_+ basic magenta system was carried out using DMPO as a spin-trapping agent. The results are shown in Figure 6b in which the free radical concentration determines the ESR signal intensity. It can be seen that there is a clear DMPO-·OH signal (four characteristic peaks, 1:2:2:1) that appeared, which proves that ·OH is produced in the Cu/Fe-NaA + H_2_O_2_+ basic magenta system. The ESR results are consistent with the free radical-trapping experiments, indicating that the presence of ·OH leads to the degradation of basic magenta in the Cu/Fe-NaA + H_2_O_2_+ basic magenta system. Figure 6c illustrates the dominant role of ·OH in the Fenton-like reaction for the degradation of basic magenta and the removal of TOC.

The possible mechanism of the Cu/Fe-NaA zeolite-like Fenton-catalyzed degradation of basic magenta is deduced and is shown in Scheme 2. The possible mechanism is as follows:

1. Initial reaction: When the Cu/Fe-NaA zeolite encounters H_2_O_2_, Cu/Fe catalysts decompose H_2_O_2_ into hydroxyl radicals (·OH). These hydroxyl radicals can oxidize organic compounds and thus catalyze to degrade basic magenta.
2. Catalytic reaction: On the surface of the Cu/Fe-NaA zeolite, Fe^3+^ is reduced to Fe^2+^, and then Fe^2+^ is oxidized back to Fe^3+^ by H_2_O_2_; Cu^2+^ is reduced to Cu^+^, and then Cu^+^ is oxidized back to Cu^2+^ by H_2_O_2_, and at the same time, hydroxyl radicals are generated. These hydroxyl radicals can degrade organic dyes on the catalyst surface.
3. Ion exchange: The sodium ions (Na^+^) of the Cu/Fe-NaA zeolite will exchange with the dye molecules of basic magenta, which can immobilize the basic magenta on the surface of the catalyst and make it easier to degrade.

Therefore, the Cu/Fe-NaA zeolite-based Fenton catalysts degrade basic magenta mainly through hydroxyl radical oxidation and ion-exchange mechanisms.

### 3.8 Recycling of the Catalyst

**Figure 7:**
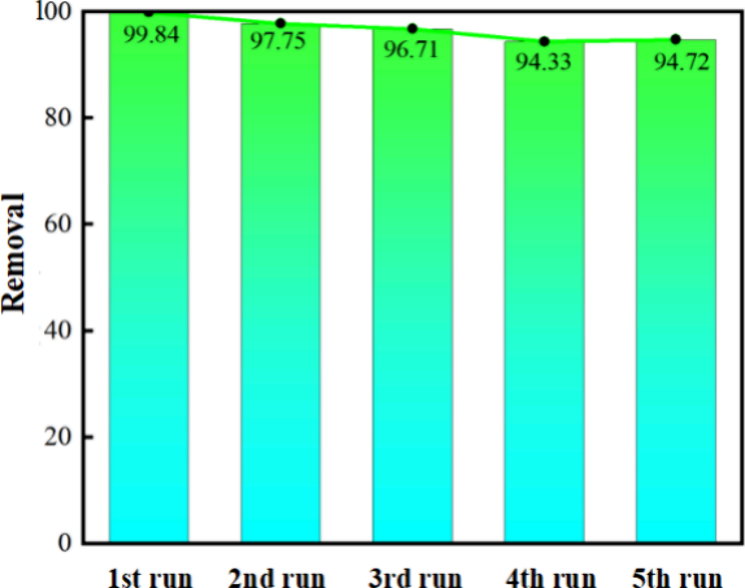
Cyclic experiments of the Cu/Fe-NaA catalyst.

The stability and reusability of the catalyst was investigated by recycling the Cu/Fe-NaA catalyst after the initial degradation of basic magenta. The reused product was used as a catalyst to repeat the basic magenta degradation process several times. A mixed solution of 6% NaOH+1% NaCl was chosen as the regeneration agent. The metal ions adsorbed in the zeolite pores would be attracted by OH^–^ under alkaline condition, and the Na^+^ in the solution could exchange the metal ions on the adsorption sites on the zeolite for the purpose of regeneration. The excellent reusability of the catalyst could be attributed to the structural stability and negligible loss of copper in the solution during the Fenton-like degradation of basic magenta. The degradation rate decreased with increasing recycling time from 99.84% (first) to 94.72% (fifth) as shown in Figure 7, indicating that the catalyst has good stability and reusability. This is in accordance with the purpose of green catalysis.

## 4 Conclusions

A highly efficient and stable zeolite catalyst (Cu/Fe-NaA) was prepared from coal gangue by the impregnation calcination method and applied in nonhomogeneous Fenton reaction for the removal of basic magenta from wastewater. Compared with NaA zeolites loaded with only one metal, Cu/Fe-NaA zeolites showed superior catalytic activity in the Fenton process and showed good catalytic performance in degrading basic magenta. The dye concentration of 250 mg/L, 40 °C, catalyst dosage of 0.6000 g, pH = 6, copper iron loading of 1.0 g/L, and 1.5 mL of H_2_O_2_ were chosen as the best conditions for catalytic degradation, and at these conditions, the catalytic degradation rate was up to 99.84%. Moreover, the catalyst shows good recyclability and stability. Therefore, Cu/Fe-NaA/H_2_O_2_ system shows great prospect in industrial application, and it promotes the way of waste conversion to a valuable resource for environmental governance.

## References

[ref1] Qi G. Progress in the preparation of gangue-based zeolite and its application in the field of environmental protection. Coal Chem. 2023, 46 (7), 110–114.

[ref2] Xu H. Y.; Yuan X. Q.; Dai L. Y.; Wang W. S.; Li Y.; Dong L. M. Fenton-like degradation of Rhodamine B over Fe_2_O_3_–Al_2_O_3_-zeolite hybrids derived from coal fly ash. International journal of Environmental Science and technology. 2023, 20 (12), 13233–13244. 10.1007/s13762-023-04884-y.

[ref3] Ting Z.; Yu S.; Ting J. Degradation of basic magenta dyes by chlorine dioxide solid formulations. Contemp. Chem. Ind. 2023, 52 (10), 2341–2344.

[ref4] Liu W.; Yin D.; Guan X.; Rao D.; Cao G.; Sun Y. Role of pyrophosphate on the degradation of sulfamethoxazole by permanganate combined with different reductants: Positive or negative. Water Environment Research. 2020, 92 (4), 604–611. 10.1002/wer.1256. 31602733

[ref5] Fida H.; Zhang G.; Guo S.; Naeem A. Heterogeneous Fenton degradation of organic dyes in batch and fixed bed using La-Fe montmorillonite as catalyst. J. Colloid Interface Sci. 2017, 490, 859–868. 10.1016/j.jcis.2016.11.085. 28002774

[ref6] Du J.; Bao J.; Fu X.; Lu C.; Kim S. H. Mesoporous sulfur-modified iron oxide as an effective Fenton-like catalyst for degradation of bisphenol A. Applied Catalysis B: Environmental. 2016, 184, 132–141. 10.1016/j.apcatb.2015.11.015.

[ref7] Clarizia L.; Russo D.; Di Somma I.; Marotta R.; Andreozzi R. Homogeneous photo-Fenton processes at near neutral pH: A review. Applied Catalysis B: Environmental. 2017, 209, 358–371. 10.1016/j.apcatb.2017.03.011.

[ref8] Ming X.; Wen X.; Chen D.; Yao B.; Jing Z.; Yi Z.; Jin Z.; Ya Y. Metal sulfides as excellent co-catalysts for H_2_O_2_ decomposition in advanced oxidation processes. Chemosphere 2018, 6, 1359–1372. 10.1016/j.chempr.2018.03.002.

[ref9] Zhang Q.; Wang Q.; Wang S. Efficient heterogeneous Fenton-like catalysis of Fe-doped SAPO-44 zeolite synthesized from bauxite and rice husk. Chem. Phys. Lett. 2020, 753, 137598 10.1016/j.cplett.2020.137598.

[ref10] Reyhani A.; Mazaheri O.; Alivand M. S.; Mumford K. A.; Qiao G. Temporal Control of RAFT Polymerization via Magnetic Catalysis. Polymer Chemietry. 2020, 11, 2838–2846. 10.1039/D0PY00220H.

[ref11] Li X.; Wu M.; Wang L.; Chen R.; Wei Y.; Liu H. In-situ generation of multi-homogeneous/heterogeneous Fe-based Fenton catalysts toward rapid degradation of organic pollutants at near neutral pH. Chemosphere 2020, 245, 125663 10.1016/j.chemosphere.2019.125663. 31877454

[ref12] Liu Y.; Zhao Y.; Zhang G.; Zhang W. Enhanced catalytic degradation of methylene blue by alpha-Fe_2_O_3_/graphene oxide via heterogeneous photo-Fenton reactions. Appl. Catal. B: Environ. 2017, 206, 642–652. 10.1016/j.apcatb.2017.01.075.

[ref13] Nuan Z.; Jun C.; Zhan F.; Eric P. T. Ceria accelerated nanoscale zerovalent iron assisted heterogenous Fenton oxidation of tetracycline. Chem. Eng. J. 2019, 369, 588–599. 10.1016/j.cej.2019.03.112.

[ref14] Zhang X.; Guo Y.; Shi S.; Liu E.; Li T.; Wei S.; Zhao Z. Efficient and stable iron-copper montmorillonite heterogeneous Fenton catalyst for removing Rhodamine B. Chem. Phys. Lett. 2021, 776, 138673 10.1016/j.cplett.2021.138673.

[ref15] Dong Q.; Chen Y.; Wang L.; Ai S.; Ding H. Cu-modified alkalinized g-C_3_N_4_ as photo catalytically assisted heterogeneous Fenton-like catalyst. Appl. Surf. Sci. 2017, 426, 1133–1140. 10.1016/j.apsusc.2017.07.254.

[ref16] Shen Z. Y.; Ying X.; Li J. F. Enhancement of Qm in CuO-Doped Compositionally Optimized Li/Ta-Modified (Na,K)NbO_3_ Lead-Free Piezoceramics. Ceram. Int. 2012, 38 (1), S331–S334. 10.1016/j.ceramint.2011.04.113.

[ref17] Sheng Y. W.; Yan Q. H.; Chen C.; Ming Z.; Nan D.; Li X. M. Effect of Oriented Defect-Dipoles on the Ferroelectric and Piezoelectric Properties of CuO-Doped (K 0.48 Na 0.52) 0.96 Li 0.04 Nb 0.805 Ta 0.075 Sb 0.12 O 3 Ceramics. Ceram. Int. 2018, 44 (9), 10141–10146. 10.1016/j.ceramint.2018.02.233.

[ref18] Meng Q.; Du L.; Yang J.; Tang Y.; Han Z.; Zhao K.; Zhang G. Well-dispersed small-sized MnOx nanoparticles and porous carbon composites for effective methylene blue degradation. Colloids and Surfaces A: Physicochemical and Engineering Aspects. 2018, 548, 142–149. 10.1016/j.colsurfa.2018.03.064.

[ref19] Boczkaj G.; Fernandes A. Wastewater treatment by means of advanced oxidation processes at basic pH conditions: a review. Chemical Engineering Journal. 2017, 320, 608–633. 10.1016/j.cej.2017.03.084.

[ref20] Lyu L.; Han M.; Cao W.; Gao Y.; Zeng Q.; Yu G.; Hu C. Efficient Fenton-like process for organic pollutant degradation on Cu-doped mesoporous polyimide nanocomposites. Environ. Sci.: Nano 2019, 6, 798–808. 10.1039/C8EN01365A.

[ref21] Liang H.; Liu R. P.; An X. Q.; Liu H. J. Activating efficiency of iron-copper bimetallic organic framework MIL-101(Fe, Cu) toward H_2_O_2_ for degradation of dyes. Huan jing ke xue = Huan-jing kexue 2020, 41 (38), 4607–4614. 10.13227/j.hjkx.202003024. 33124393

[ref22] Yang X.; Sun H.; Zhang L.; Zhao L.; Lian J.; Jiang Q. High efficient photo-Fenton catalyst of α-Fe_2_O_3_/MoS_2_ hierarchical nano-heterostructures: reutilization for supercapacitors. Sci. Rep. 2016, 6 (1), 31591 10.1038/srep31591. 27526965 PMC4985694

[ref23] Li L.; Lai C.; Huang F.; Cheng M.; Zeng G.; Huang D.; Chen L. Degradation of naphthalene with magnetic bio-char activate hydrogen peroxide: synergism of bio-char and Fe–Mn binary oxides. Water Res. 2019, 160, 238–248. 10.1016/j.watres.2019.05.081. 31152949

[ref24] Chen T.; Zhu Z.; Zhang H.; Shen X.; Qiu Y.; Yin D. Enhanced Removal of Veterinary Antibiotic Florfenicol by a Cu-Based Fenton-like Catalyst with Wide pH Adaptability and High Efficiency. ACS Omega. 2019, 4, 1982–1994. 10.1021/acsomega.8b03406. 31459449 PMC6648108

[ref25] Li L.; Lai C.; Huang F.; Cheng M.; Zeng G.; Huang D.; Chen L. Degradation of naphthalene with magnetic biochar activate hydrogen peroxide: Synergism of biochar and Fe- Mn binary oxides. Water Res. 2019, 160, 238–248. 10.1016/j.watres.2019.05.081. 31152949

[ref26] Herney-Ramirez J.; Vicente M. A.; Madeira L. M. Heterogeneous photo-Fenton oxidation with pillared clay-based catalysts for wastewater treatment: A review. Appl. Catal. B: Environ. 2010, 98, 10–26. 10.1016/j.apcatb.2010.05.004.

[ref27] Cheng M.; Zeng G.; Huang D.; Lai C.; Liu Y.; Zhang C. Efficient degradation of sulfamethazine in simulated and real wastewater at slightly basic pH values using Co-SAM-SCS /H_2_O_2_ Fenton-like system. Water Res. 2018, 138, 7–18. 10.1016/j.watres.2018.03.022. 29558693

[ref28] Gao J.; Yu T. L.; Xin N. X.; Long L. W.; Wan D. Fe1-xZnxS Ternary Solid Solution as an Efficient Fenton-like Catalyst for Ultrafast Degradation of Phenol. J. Hazard. Mater. 2018, 353 (7), 393–400. 10.1016/j.jhazmat.2018.04.029. 29698904

[ref29] Li Q.; Wei G.; Yang Y.; Li Z.; Zhang L.; Shao L.; Lai S. Insight into the enhanced catalytic activity of a red mud based Fe_2_O_3_/Zn–Al layered double hydroxide in the photo-Fenton reaction. Catalysis Science & Technology. 2020, 10 (21), 7365–7377. 10.1039/D0CY01539C.

[ref30] Jin H.; Tian X.; Nie Y.; Zhou Z.; Yang C.; Li Y.; Lu L. Oxygen Vacancy Promoted Heterogeneous Fenton-like Degradation of Ofloxacin at pH 3.2–9.0 by Cu Substituted Magnetic Fe_3_O_4_@FeOOH Nanocomposite. Environmental Science & Technology. 2017, 51, 12699–12706. 10.1021/acs.est.7b04503. 28934546

